# P-1554. Cytokine dysregulation in Scrub typhus: Correlation with complications and disease severity

**DOI:** 10.1093/ofid/ofaf695.1734

**Published:** 2026-01-11

**Authors:** Navneet Sharma, Mohan Kumar, Manisha Biswal, Nalin Sharma, Rojar singh, Deba Prasad, Ashok Pannu, Ashish Behera, Neeraj SIngla

**Affiliations:** Postgraduate Institute of Medical Education and Research, Chandigarh, Chandigarh, India; Postgraduate Institute of Medical Education and Research, Chandigarh, Chandigarh, India; PGIMER, Chandigarh, Chandigarh, India; Mayo Clinic, Rochester, Minnesota; Postgraduate Institute of Medical Education and Research, Chandigarh, Chandigarh, India; Postgraduate Institute of Medical Education and Research, Chandigarh, Chandigarh, India; Postgraduate Institute of Medical Education and Research, Chandigarh, Chandigarh, India; Postgraduate Institute of Medical Education and Research, Chandigarh, Chandigarh, India; Postgraduate Institute of Medical Education and Research, Chandigarh, Chandigarh, India

## Abstract

**Background:**

Pathogenesis of Scrub typhus (ST), a tick-borne acute zoonotic disease caused by the bacterium *Orientia tsutsugamushi,* involves infection of vascular endothelial cells, triggering a potent cytokine host response that causes organ damage.

**Figure:**
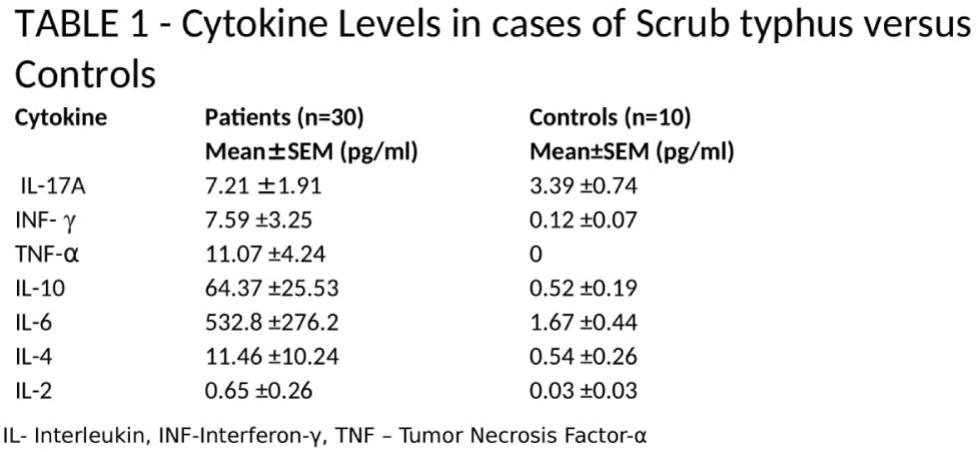

**Methods:**

Prospective study in a tertiary care hospital in North-west India in 30 cases of Scrub typhus. Measurements of cytokines IL-17A, INF-γ, TNF-α, IL-10, IL-6, IL-4, and IL-2 were carried out at admission in all 30 patients and a control group of 10 healthy persons using BD^TM^ Cytometric Bead Array, BD Biosciences, San Diego, California, USA.

**Results:**

Mean age of the entire cohort was 36 years, and the male-female ratio was 2:1. The most common symptoms were dyspnea 87%, altered sensorium 23%, abdominal pain 20%, decreased urine output 10% and myalgia in 10%. On clinical examination, eschar was present in 40%. Investigations revealed anemia in 80% cases, leukocytosis in 46.6%, thrombocytopenia in 90%, jaundice in 53.3% and elevated serum transaminase levels in 84% cases. Of all complications, acute respiratory distress syndrome occurred in 90% patients, acute kidney injury in 37% patients, acute meningoencephalitis syndrome in 30%, and myocarditis in 23%. The mean APACHE 2 score of the entire cohort was 12 . Multi-organ dysfunction syndrome (MODS) was seen in 67% of cases, and the overall mortality rate was 20%. On Fisher's Exact Correlation analysis, presentation with meningoencephalitis, myocarditis, systemic acidosis, and a high APACHE 2 score correlated with mortality. The serum levels of cytokines were elevated in all patients compared to the healthy control group (Table 1), but did not significantly differ between survivors and non-survivors. Furthermore, the elevated levels of TNF-α (p=0.004) and IL-10 (p=0.029) strongly correlated with multi-organ dysfunction syndrome (MODS).

**Conclusion:**

In Scrub Typhus, the strong correlation of serum levels of TNF-α and IL-10 with multi-organ dysfunction syndrome (MODS) suggests their involvement in disease progression. The high prevalence of acute respiratory distress syndrome and MODS, alongside a mortality rate of 20%, highlights the critical nature of scrub typhus and the necessity for early recognition of complicated disease and intervention.

**Disclosures:**

All Authors: No reported disclosures

